# Programmed death-1 expression and regulatory T cells increase in the Intestinal mucosa of cytomegalovirus colitis in patients with HIV/AIDS

**DOI:** 10.1186/s12981-020-00315-x

**Published:** 2020-09-05

**Authors:** Lei Sun, Kun Yang, Liang Zhang, Li-ming Qi, Jia-min Chen, Ping Li, Jiang Xiao, Hong-xin Zhao, Peng Wang

**Affiliations:** 1 Department of Pathology, Beijing Ditan Hospital, Capital Medical University, No. 8 Jing Shun East Street, Chaoyang District, Beijing, 100015 People’s Republic of China; 2 Department of Gastroenterology, Beijing Ditan Hospital, Capital Medical University, Beijing, 100015 China; 3 Center for Infectious Diseases, Beijing Ditan Hospital, Captial Medical University, Beijing, 100015 China

**Keywords:** HIV/AIDS, CMV colitis, Intestinal mucosa, Regulatory T cells (T_regs_), PD-1

## Abstract

**Background:**

Cytomegalovirus (CMV) is among the most common opportunistic infections identified in patients with HIV/AIDS. CMV often targets the colon in such patients. However, the role of regulatory T cells (T_regs_) and Programmed death-1 (PD-1) in intestinal CMV infection is unclear. In this study, we evaluate the expression of programmed death -1 (PD-1) and its association with regulatory T cells (T_regs_) in patients with HIV/AIDS having CMV colitis.

**Methods:**

CMV was detected in the intestinal mucosal biopsy samples via nucleic acid in situ hybridization. PD-1, CD4, CD8, and T_reg_-specific marker as well as the winged-helix transcription factor and forkhead box P3 (FoxP3) were detected by immunohistochemical methods.

**Results:**

Intestinal CMV diease was identified in 20 out of 195 patients with HIV/AIDS enrolled in our study. CMV was diagnosed microscopically by the presence of giant cell inclusion bodies in epithelial cells, histiocytes, and fibroblasts. Levels of immunoreactive PD-1 detected in mucosal biopsies from patients with HIV/AIDS having CMV colitis were significantly higher than CMV-negative control group (*p* = 0.023). FoxP3^+^ cells were detected in the CMV colitis group slight more than that in the control group. CD4^+^ T lymphocyte counts in the peripheral blood and intestinal mucosal biopsies from CMV colitis group were all notably decreased compared with those with control group (*p* < 0.001 for both). PD-1 had a significant negative correlation with CD4 counts in intestinal mucosa (*p* = 0.016). CD8^+^T lymphocyte counts in peripheral blood and intestinal mucosa were slightly lower than those in the control group, although the differences were not statistically significant.

**Conclusions:**

CMV colitis with HIV/AIDS is associated with significant changes in T lymphocyte populations. These findings may have important implications for disease pathogenesis and progression.

## Background

CMV is the among most common pathogen causing opportunistic infections detected in patients with infections caused by human immunodeficiency virus/acquired immunodeficiency syndrome (HIV/AIDS) [1]. The reactivation of CMV occurs more frequently in immunocompromised patients. In recent years, in China, the number of patients with HIV/AIDS has increased, and the incidence of CMV infection in such patients is increasing every year. CMV infections in the intestine are common in these patients. CMV can infect epithelial cells, histiocytes, fibroblasts, smooth muscle cells etc., which could result in a lytic infection that leads to necrosis, erosion, mucosal ulceration and bleeding.

A variety of immune mechanisms play an important role in viral infections, such as the induction of regulatory T cells (T_regs_) and expression of immune checkpoint molecule Programmed death 1 (PD-1) [2].Persistent exposure to viral antigens can lead to high PD-1 expression and T cell exhaustion [3]. Several studies have suggested that T_regs_ contribute to viral persistence in human and mouse chronic infections [4], as well as PD-1 plays an important role in the activation of T_regs_ in HIV infection [5].However, the changes of T_regs_ and PD-1 in intestinal CMV infection is unknown.

T_regs_ inhibit innate and acquired immune function and can modulate anti-microbial immunity; they also reduce immune damage and maintain immune tolerance. The transcription factor forkhead box P3 (FoxP3) is expressed only in T_regs_ and is a specific marker for this lymphocyte lineage [6].

PD-1 is a negative costimulatory receptor and member of the immunoglobulin superfamily that has been widely expressed on activated T lymphocytes, natural killer cells, and myeloid cells [7]. PD-1 plays an important role in modulating tumor immune evasion, immune tolerance, autoimmune diseases, and host responses to bacterial and viral infections by transmitting negative stimuli [8]. Moreover, signaling via PD-1 and its ligand PD-L1 also inhibits the proliferation and activation of T cells by regulating the secretion and expression of proinflammatory cytokines and regulates the function of T_regs_ [9].

In this study, we detected FoxP3 and PD-1 in intestinal mucosal biopsies of patients with HIV/AIDS both with and without concomitant CMV colitis.

## Methods

### Patients

Overall, 195 patients with HIV/AIDS were enrolled in our study at Beijing Ditan Hospital from January 2010 to January 2019. Among these, 173 were male, and 22 were female; all were within the age range of 10 to 73 years old. The average age was 38.0 ± 11.6 years. All patients who were enrolled in the study had reached the AIDS stage and did not start ART prior to recruitment. They underwent colonoscopy to evaluate gastrointestinal symptoms, including abdominal pain, diarrhea, and blood in the stool. Some patients had opportunistic infections or malignancies in other organs outside the gastrointestinal tract. Based on colonoscopy and mucosal biopsy findings, patients with HIV/AIDS were divided into two groups, the first with cytopathological evidence of CMV colitis and a second group with non-specific gastrointestinal inflammation. Clinical information such as demographic profile and serological testing was obtained from the patients’ charts through our electronic medical record system. Written informed consent was obtained from the participants by physicians. The study protocol was approved by the Ethics Committee of Beijing Ditan Hospital, Capital Medical University.All procedures performed in studies involving human participants were in accordance with the ethical standards and with the 1964 Helsinki declaration and its later amendments or comparable ethical standards.

### Colonoscopy and acquisition of intestinal mucosal tissue specimens

Patients underwent regular bowel preparation prior to colonoscopy. As part of the procedure, mucosal tissue from inflammatory foci in the rectum, sigmoid, colon and cecum were sampled together with regions that appeared to be unaffected. Tissues were fixed in 10% formaldehyde solution. Serial sections. (4 μm) of formalin-fixed, paraffin-embedded tissue were subjected to hematoxylin and eosin (HE), acid-fast, hexamine silver, and periodic acid-Schiff staining. Samples were also prepared for immunohistochemistry and detection of CMV by nucleic acid in situ hybridization.

### Reagents

Anti-human FoxP3 monoclonal antibody (mAb) was from Abcam Company (Clone number: 236A/E7, Lot: GR45435-1). Anti-human PD-1 mAb was from Gene Tech Company (Clone number: 2E5, Lot:GT228102), and anti-human CD4 (Clone number:UMAB64, Lot: ZM-0418) and anti-human CD8 (Clone number:SP16, Lot: ZM-0508) antibodies were from Beijing Zhongshan Biotechnology. Horseradish peroxidase-labeled secondary antibodies and 3,3′-diaminobenzidine (DAB) chromogenic kit were also from Beijing Gene Company. A hybridization kit containing a digoxigenin probe for detection of CMV and a nitro blue tetrazolium (NBT)/5-bromo-4-chloro-3-indolyl phosphate (BCIP) assay kit were from Leica Biosystems (CMV Probe:REF:PB0614, Lot:62163).

### EnVision two-step method for immunohistochemical staining

Embedded sections were deparaffinized with xylene followed by an alcohol gradient and water rinses and incubated with 0.3% hydrogen peroxide for 10 min at room temperature to eliminate endogenous peroxidase activity. After antigen retrieval under high pressure with a citrate buffer, individual slides were incubated at 4 °C overnight with mouse anti-human FoxP3 (1:40 dilution), mouse anti-human PD-1 (1:100 dilution), rabbit anti-human CD4 (1:100 dilution), or rabbit anti-human CD8 (1:100 dilution), Slides were then washed three times with phosphate buffered saline (PBS), followed by horseradish peroxidase-labeled secondary antibodies (37 °C for 30 min). Slides were then washed and developed with DAB, hematoxylin counterstained, and mounted. PBS diluent was used in place of individual primary antibodies as negative controls.

### CMV nucleic acid in situ hybridization

Nucleic acid in situ hybridization using digoxigenin-labeled probes was conducted according to the manufacturer’s instructions. The dilution of the CMV probe was 1:50–1:100, which generated a strong brown-to-yellow positive signal.

### Evaluation of immunohistochemistry

Three regions of the lamina propria were selected at random for microscopic evaluation. Under high magnification (Nikon 80i, ×400), a grid counter was used to assist with enumeration of FoxP3^+^ (T_regs_), CD4^+^ T lymphocytes, CD8^+^ T lymphocytes, and total PD-1^+^ lymphocytes. Data generated were presented as percentages: the number of specific antigen positive cells × 100/total number of lymphocytes detected in the grid.

### Statistical methods

All statistical tests were performed using SPSS (IBM statistics, Version 20.0, SPSS, Chicago, IL). Values are expressed as mean ± standard deviation (SD). Descriptive statistical analyses (median, 25th and 75th percentile) were used to summarize patient characteristics. Continuous variables were tested for normality. Differences between CMV colitis and non CMV colitis patients with HIV/AIDS were evaluated using Student’s t test. Categorical variables were analyzed using Chi square test. Spearman’s correlation analysis were used to study the correlation between PD-1 and CD4 counts. Continuous data are presented as mean ± standard deviation (SD); *p* values of < 0.05 were considered significant.

## Results

### Patient characteristics

Of the 195 patients with HIV/AIDS enrolled in this study, we diagnosed 20 (10.3%) with CMV colitis. Of the original 173 male patients, 18 (10.4%) were diagnosed with CMV colitis. Similarly, of the original 22 female patients, 2 (9%) were found to have CMV colitis; we detected no difference in distribution by sex (*p* = 0.848). The average age of patients with HIV/AIDS having CMV colitis was 39.1 ± 11.2 years, which was not significantly higher than that of those in the control group (37.9 ± 11.6 years; *p* = 0.666). Patient demographic information is presented in Table 1.

**Table 1:** Comparison of demographic profile and serological testing between HIV/AIDS patients with and without CMV colitis

	HIV+ CMV (CMV colitis group, n = 20) (mean ± SD; Median; IQR)	HIV (control group, n = 175) (mean ± SD; Median; IQR)	Total	P value
Sex				
Male	18 (90%)	155 (88.6%)	173 (88.7%)	0.848
Female	2 (10%)	20 (11.4%)	22 (11.3%)	
Age (year)				
Average	39.1 ± 11.2; 41; 27–51	37.9 ± 11.6; 35; 29–46	38.0 ± 11.6;36; 29–46	0.666
Serum CD4+ T cells (cells/μL)	31.3 ± 40.3; 14; 5–46	210.6 ± 217.1; 140.5; 24.5–345.5;	–	< 0.001
Serum CD8+ T cells (cells/μL)	531.6 ± 500.6; 468; 123–682	746.3 ± 543.1; 617.5; 386.25–918.75	–	0.102
Leukocyte (10^9^/L)	5.08 ± 4.51; 3.77; 2.25–5.39	5.21 ± 2.61; 4.84; 3.245–6.475	–	0.902
Erythrocyte (10^12^/L)	2.91 ± 0.54; 2.81; 2.47–3.4	3.71 ± 0.96; 3.74; 2.97–4.47	–	< 0.001
Hemoglobin (g/L)	87.3 ± 17.9; 88.4; 72.2–107	115.7 ± 30.7; 115; 94–141.1	–	< 0.001
Platelet (10^9^/L)	189.4 ± 120.3; 173; 118–221.8	207.9 ± 83.5; 205.5; 154.95–261.5	–	0.385
HIV viral load (copies/mL)	261,939 ± 241,476; 302,918; 53,904–387,750	337,875 ± 971,601; 90,565.5; 11,341.25–441,623	–	0.764

### Serological testing of patients with HIV/AIDS

Compared with the control group, the number of CD4^+^ T lymphocytes in peripheral blood in the CMV colitis group was significantly reduced; the number of red blood cells and hemoglobin content were also significantly lower (*p* values all < 0.001, Table 1).Among 20 patients with CMV colitis, blood CMV DNA was positive in 15 cases (75%). Serologic detection of CMV antibodies IgG and IgM were positive in 4 (20%) and 1 (5%) cases, respectively.

### Pathologic findings and cellular localization of CMV in mucosal biopsies from patients with HIV/AIDS with CMV infection

**Fig. 1:**
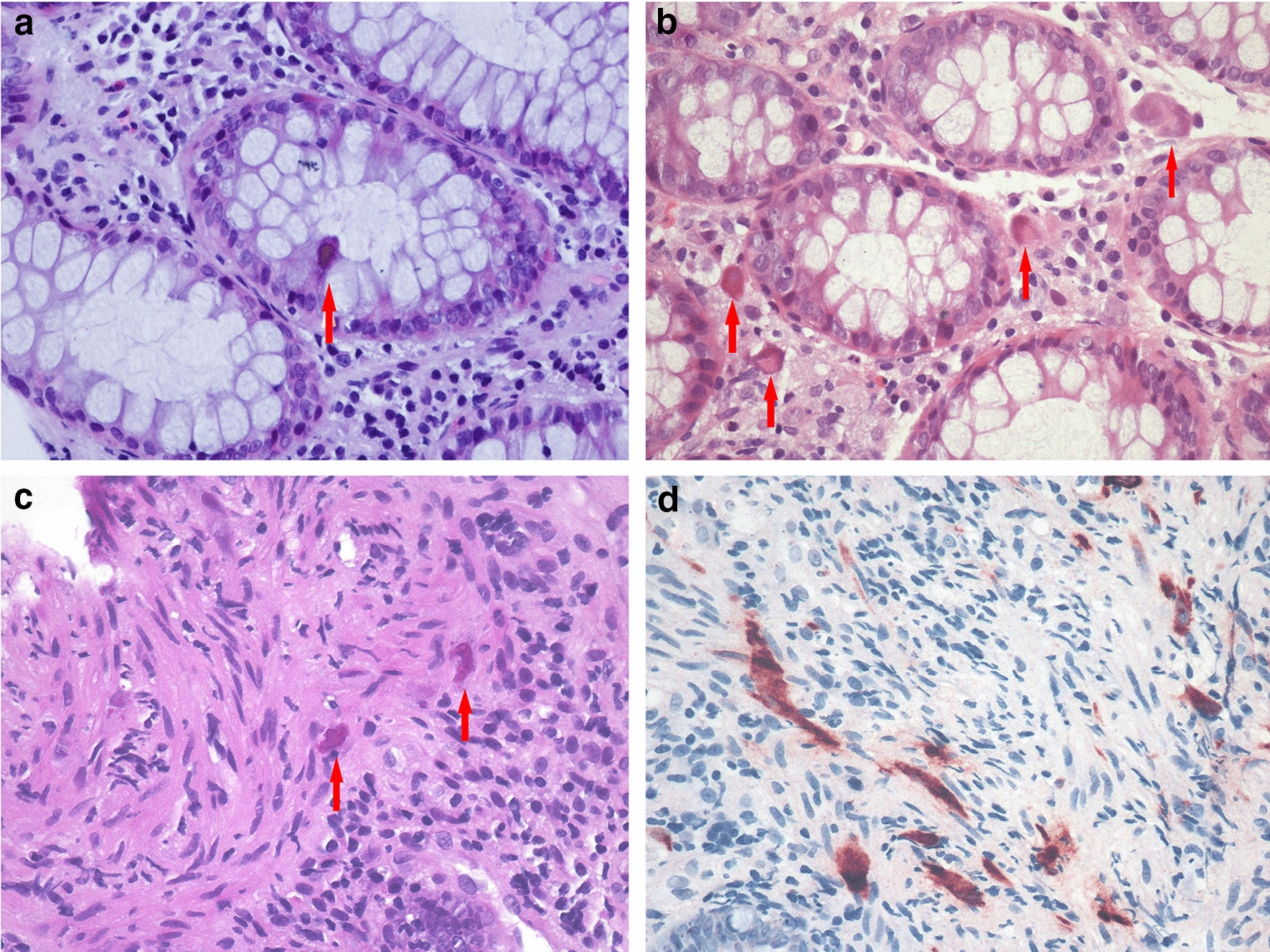
Cellular localization of CMV in the intestinal mucosa of patients with HIV/AIDS with CMV colitis. Formation of giant cell inclusion bodies (arrows) in epithelial cells (**a**), histiocytes (**b**),and fibroblasts (**c**) (HE, 400×); In situ hybridization of CMV in fibroblasts shows a brown positive signal (D) (NBT/BCIP, 400×)

Characteristics of the intestinal mucosa biopsy samples included edema, congestion, lymphocytosis, and rare lymphoid aggregates. Some areas of ulceration and granulation tissue were also detected (Table 2). H&E staining and nucleic acid in situ hybridization for CMV revealed giant cell inclusion bodies in epithelial cells, histiocytes and fibroblasts; the inclusion bodies were round or oval and were surrounded by a distinct air halo, a characteristic also known as the “owl’s eye.” Other CMV inclusions were characterized by abundant coarse eosinophilic cytoplasmic inclusions within enlarged cells without clear nuclear inclusions (Fig. 1).

**Table 2:** Pathologic findings in mucosal biopsies from patients with HIV/AIDS with CMV infection

Pathological changes	Edema	Congestion	Lymphocyte infiltration	Neutrophil infiltration	Ulceration	Granulation	nuclear inclusion “owl’s eye”	Cytoplasmic inclusion	Total
Number of cases	16 (80%)	6 (30%)	20 (100%)	11 (55%)	10 (50%)	2 (10%)	5 (25%)	17 (85%)	20 (100%)

### FoxP3, PD-1, and CD4^+^ T cells in intestinal mucosa

**Fig. 2:**
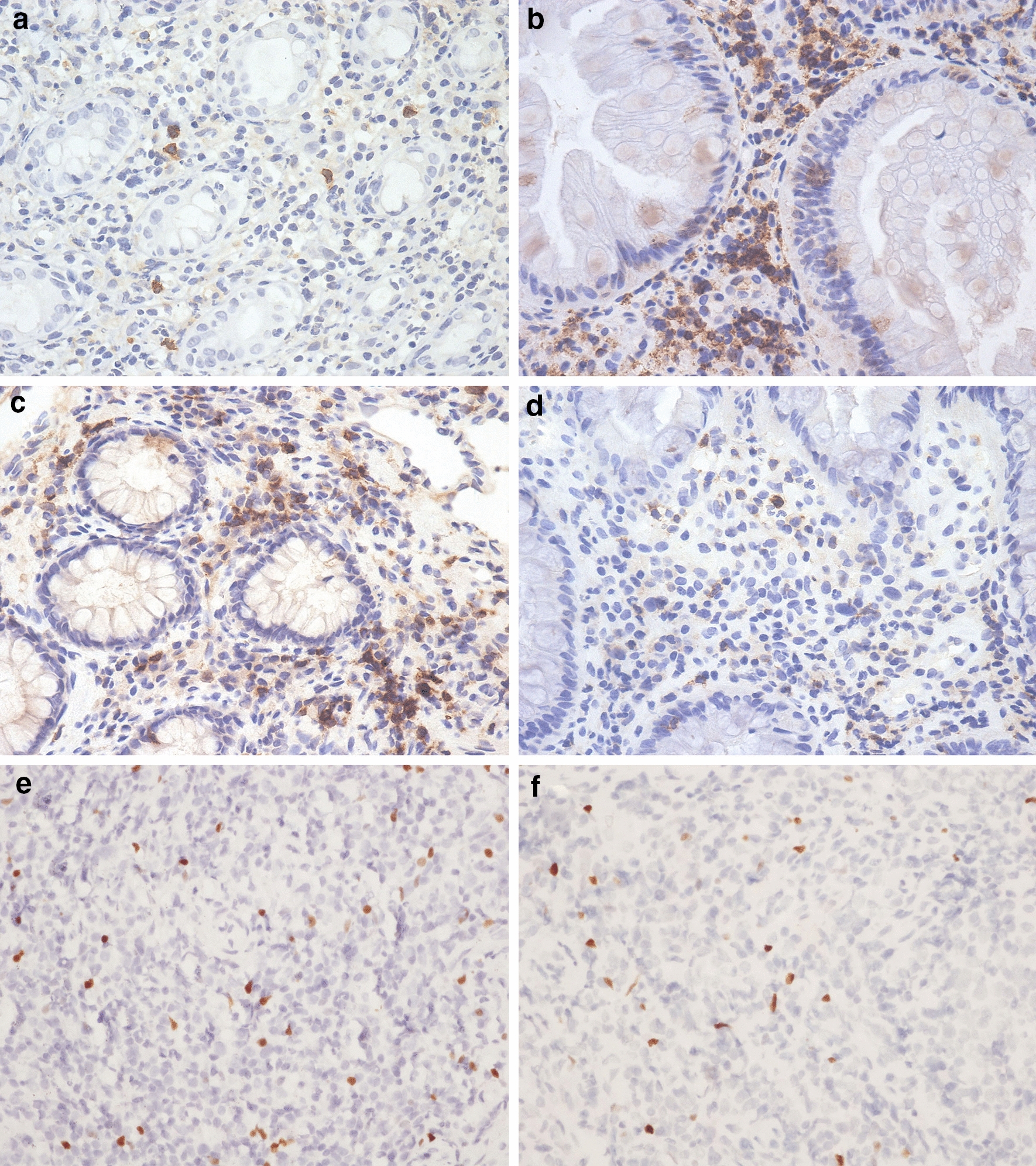
CD4,PD-1 and FoxP3 positive cells were mainly distributed in the intestinal lamina propria. CD4 (**a**, **b**) and PD-1 (**c**, **d**) were detected primarily at the cell membranes and within the cytoplasm (IHC, ×200), whereas FoxP3 was localized in the nucleus (**e**, **f**) (IHC, ×200). HIV+ CMV+ patients (**a**, **c**, **e**), HIV+ CMV-patients (**b**, **d**, **f**)

CD4^+^ T cells as well as FoxP3^+^ and PD-1^+^ cells were mainly distributed within the intestinal lamina propria. Immunoreactive CD4 and PD-1 were detected primarily at the cell membranes and within the cytoplasm, whereas FoxP3 was localized in the nucleus (Fig. 2).

**Fig. 3:**
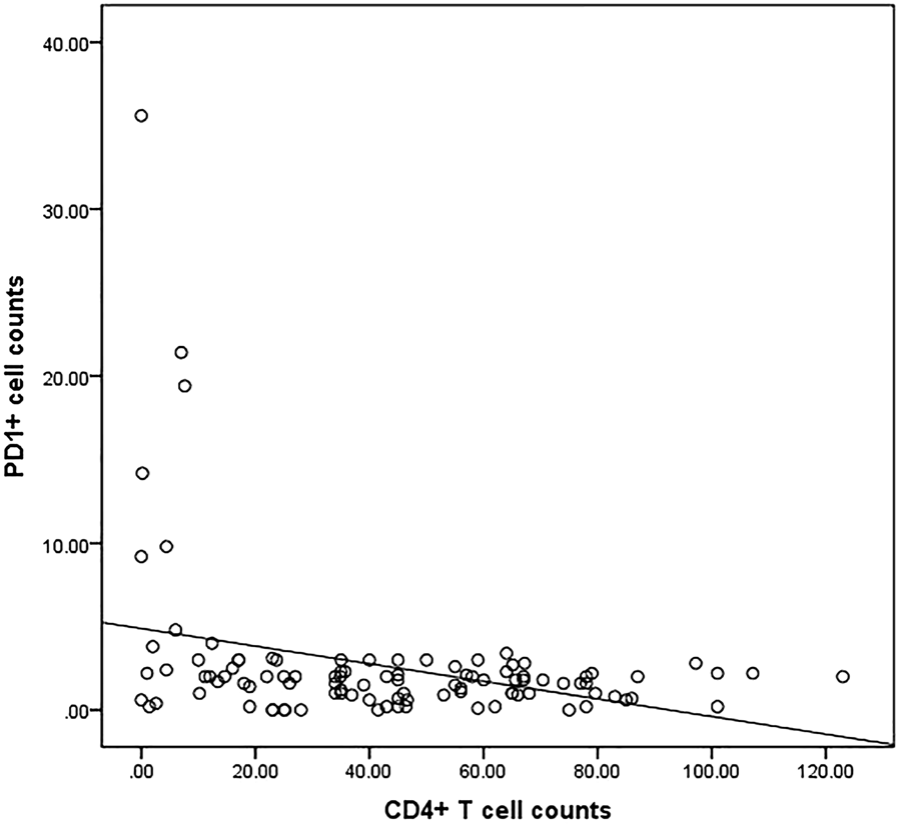
PD-1 had a significant negative correlation with CD4 counts in intestinal mucosa in patients with HIV/AIDS (r_s_ = − 0.238, P = 0.016)

In the intestinal mucosa of patients with HIV/AIDS having CMV colitis, 3.1% ± 3% of the total lymphocytes were CD4^+^. This fraction was significantly lower than the 14.9% ± 7% CD4^+^ T cells detected in samples from the control group (*p* < 0.001); The fraction of PD-1^+^ cells in the intestinal mucosa of the CMV colitis group was significantly higher than that identified in the control group (9.0% ± 10.8% vs. 1.3% ± 1.1%, *p *= 0.023). The PD-1 expression level in intestinal mucosa of HIV/AIDS patients was significantly negative correlated with the number of CD4 + T cells (r_s_ = − 0.238, *p* = 0.016) (Fig. 3). Furthermore, in the CMV colitis group, the fraction of FoxP3^+^ cells showed an increasing trend compared with the control group, although the difference was not statistically significant (*p *= 0.156). Finally, the fraction of CD8^+^ T cells in the CMV colitis group was lower than that detected in the control group, with no statistically significant difference (Table 3).

**Table 3:** The positive label index of CD4, CD8, Fox-P3,PD-1 in intestinal mucosa

Test items group	HIV + CMV (n = 20)	HIV (n = 175)	P value
Intestinal mucosal CD4+ T cells	3.1% ± 3.3%	14.9% ± 6.8%	< 0.001
Intestinal mucosal CD8+ T cells	12.45% ± 6.81%	18.22% ± 8.69%	0.252
Intestinal mucosal Fox-P3+ cells	1.10% ± 0.32%	0.81% ± 0.31%	0.156
Intestinal mucosal PD-1+ cells	9.0% ± 10.8%	1.3% ± 1.1%	0.023

In addition, among 20 patients with CMV colitis, there was no significant difference in CD4 and PD-1 expression between blood CMV DNA positive and negative patients (*p *= 0.376 and 0.677, respectively), that between serum IgG positive and negative patients was also no significant difference (*p *= 0.281 and 0.159, respectively).

## Discussion

CMV is a double-stranded DNA virus belonging to the β-herpesvirus family. The virus is widespread in the general population; CMV seroprevalence may be > 60% [10]. CMV infection can be initiated through close contact, intravenous injection, blood transfusion, sexual intercourse, placental transmission, or organ transplantation. Primary CMV infection is subclinical in most healthy individuals. However, CMV is among the most common opportunistic infections in patients with HIV/AIDS and is a recognized cause of gastrointestinal infection in patients with advanced AIDS [11]. Among patients with HIV/AIDS, the risk of CMV infection increases in association with diminished CD4^+^ T-cell counts. CMV gastrointestinal infection may be detected in any region of the bowel but typically affects the colon and often manifests with diarrhea. Within the gastrointestinal tract, CMV colitis is generally characterized by mucosal ulceration associated with hemorrhage [12], which may result in decreased levels of red blood cells and hemoglobin. Compared with the control group, the number of red blood cells and hemoglobin content were also significantly reduced in patients with HIV/AIDS having CMV colitis in this study. The diagnosis of CMV colitis is established pathologically by the identification of viral cytopathic effect (CPE) in biopsy tissue. Typically, CPE includes intranuclear or cytoplasmic viral inclusions in epithelial cells, histiocytes, and fibroblasts in the intestinal mucosa.

Serological methods indirectly provide evidence of CMV infection by detection of antibodies in serum. However, Serologic detection of CMV IgM and IgG are not often helpful in identifying active disease but may determine current or prior exposure [13]. CMV seroprevalence can vary from 40 to 100% in the adult population depending on age, different geographic regions and economic status [14]. it’s not uncommon to have GI CMV disease without postive CMV antibodies and viremia [13]. We found Only 20% of patients with CMV colitis had CMV-specific IgG antibody in their serum, which may be due to the impaired humoral response to CMV in these AIDS patients. Therefore, the roles of serologic testing appears to be limited in the diagnosis of CMV colitis in AIDS.

Chronic HIV infection is characterized by the depletion of CD4+ T cells and dysfunction of effector CD8+ T cells. Prolonged antigen exposures during infections give rise to T-cell exhaustion, therefore, it is important to maintain a balance of immune response and tolerance during HIV infection. Regulatory T cells (T_regs_) are uniformly FoxP3^+^ and are essential for maintaining immune homeostasis, preventing autoimmunity, and regulating chronic inflammatory diseases [15]. In patients with HIV/AIDS who are not receiving anti-retroviral therapy, the proportion of T_regs_ increases in correlation with disease progression [5].

In this study, we detected both FoxP3^+^T_regs_ together with CMV in intestinal mucosal biopsies of patients with HIV/AIDS. Our results showed an increase in intestinal T_regs_ in the CMV colitis group compared with that in the control group, although the difference was not statistically significant that may be due to small number of CMV colitis. Whereas CD4^+^ lymphocyte counts in peripheral blood and intestinal mucosa were all significantly decreased. CMV infection in patients with HIV/AIDS may be associated with increasing numbers of T_regs_, suggesting that CMV infection promotes the production of T_regs_; T_regs_ inhibit the production of mature dendritic cells and other antigen-presenting cells, contributing to CD4^+^ and CD8^+^ T lymphocyte dysfunction, which serves to aggravate the damage of cellular immune function, eventually leading to death. Inhibition of T_regs_ combined with effective antiviral treatment may be a beneficial therapy for long-term prognosis of patients with HIV/AIDS.

We also detected PD-1, a negative regulatory factor involved in maintaining immune homeostasisin patients with HIV/AIDS. PD-1 and its receptor PD-L1/PD-L2 are negative costimulatory molecules. These two ligands which interact with PD-1 on activated T cells induce the negative inhibitory effects on T cell function, resulting in the down regulation of the immune response [16]. the PD-1/PD-L1 signaling pathway limits the immune function of T lymphocytes and has an impact on the secretion of critical cytokines, including IL-2. Recently, there have been several reports on the up-regulation of PD-1 expression in response to infection, confirming that the PD-1 signaling pathway is involved in infectious disease processes [17–19]. Expression of PD-1 is enhanced on both CD4^+^ and CD8^+^ T cells in peripheral blood of patients with HIV/AIDS compared with healthy controls and that expression of PD-1 by T_regs_ directly correlates with disease progression [20, 21]. In this study, immunohistochemical methods were used to detect PD-1 in the intestinal mucosa of patients with HIV/AIDS; CD4^+^ T cells were also enumerated. Taken together, our results showed that the expression of PD-1 was significantly higher in the CMV colitis group than that in the control group, whereas the fraction of CD4^+^ T cells showed the opposite trend. Interestingly, expression of PD-1 correlated negatively with fraction of CD4^+^ T cells, which was consistent with our understanding of its role as a sign of HIV infection progression. In addition to its role in inhibiting effector T-cell responses, the PD-1–PD-L1 interaction has been implicated in promoting induction of T_regs_ and decreasing their rate of apoptosis [2, 22]. We speculated that PD-1 may be an activation-induced inhibitor that functions to up-regulate T_regs_ and limit T-cell responses in patients with HIV/AIDS having CMV colitis; it is interesting to consider the fact that immune PD-1 mediated checkpoint blockade might be beneficial in these patients. Indeed, there are at least two published studies that suggest that immune checkpoint inhibitors may be beneficial in enhancing T-cell responses to patients with HIV [23, 24].

## Conclusions

Our findings suggested that CMV colitis associated with HIV/AIDS is associated with significant changes in T lymphocyte populations that are critical for understanding disease pathogenesis. Due to limited markers assessed, more studies will be required to determine the therapeutic benefit of immune checkpoint blockade in patients with HIV/AIDS alone or in association with CMV colitis.

## Data Availability

The datasets used and/or analyzed during the current study available from the corresponding author on reasonable request.
